# Phenotypic divergence among the members of the African malaria mosquitoes and strategies of persistence throughout the dry season

**DOI:** 10.1186/1475-2875-13-S1-O2

**Published:** 2014-09-22

**Authors:** Tovi Lehmann, Adama Dao, Alpha S Yaro, Diana L Huestis, Moussa Diallo, Seydou Timbiné, Yaya Kassogué, Alpha Adamou, Adama I Traoré, Djibril Samaké

**Affiliations:** 1 Laboratory of Malaria and Vector Research, NIAID, NIH, Rockville, MD, 20852, USA; 2 Mali International Center for Excellence in Research (ICER), University of Sciences, Techniques and Technologies, Point G, Bamako, Mali

## 

Genetic heterogeneity in the African malaria mosquito, *Anopheles gambiae*, led to the recognition of chromosomal forms over 30 years ago by Prof Coluzzi and colleagues, who proposed that divergence between these populations was extensive and related to adaptation to mesic vs arid habitats as well as to particular types of larval habitats. Re-defined as “molecular forms,” genetic divergence among these populations has been elaborated by cutting-edge molecular studies, providing support for their status as distinct evolutionary units as well as against it, and fueling a lively debate that recently culminated in the elevation of the M and S forms to species, as *A. coluzzii* and *A. gambiae*, respectively. However, beyond geographical and seasonal observations, phenotypic differences between the forms have received far less attention. Surprisingly, minimal differences were found in adult body size, reproductive output, wet-season longevity, susceptibility to *Plasmodium*, desiccation resistance, and high-temperature tolerance; together, these results appear to undercut the implications of genetic divergence. Yet, evidence for differential predator-mediated developmental success of larvae supported adaptive divergence to distinct larval habitats, while additional studies showed that reproductive isolation across most of their ranges is maintained primarily by swarm segregation and possibly by within-swarm recognition based on flight pattern, sound, or chemical cues. More recently, studies have revealed striking divergence in dry-season persistence strategies between Sahelian populations of these species. Accordingly, *A. coluzzii* persists through the dry season *in situ*, by a form of dormancy (aestivation), whereas *A. gambiae* recolonizes the area by wind-assisted long-distance migration. These results reveal profound ecological (behavioral and physiological) divergence between these populations, lending substantial support for their status as distinct species. Additionally, they affirm Prof Coluzzi’s insights about the evolutionary forces that have led to their speciation. Although these findings provide strong clues as to the process of speciation in this complex, important gaps in this understanding remain, and hold promise for fresh scientific insights and for malaria control.

